# Association between breast diseases and symptomatic uterine fibroids by using South Korean National Health Insurance database

**DOI:** 10.1038/s41598-023-43443-w

**Published:** 2023-10-05

**Authors:** Jin-Sung Yuk, Seung-Woo Yang, Sang-Hee Yoon, Myoung Hwan Kim, Yong-Soo Seo, Yujin Lee, Yilseok Joo, Jungbin Kim, Sam-Youl Yoon, Hyunjin Cho, Keunho Yang, Geumhee Gwak

**Affiliations:** 1 Department of Obstetrics and Gynecology, Sanggye Paik Hospital, School of Medicine, Inje University, Seoul, Republic of Korea; 2 Department of Surgery, Sanggye Paik Hospital, School of Medicine, Inje University, 1342, Dongil-ro, Nowon-gu, Seoul, 01757 Republic of Korea

**Keywords:** Cancer, Endocrinology, Medical research, Oncology

## Abstract

Both the uterus and breasts have sex hormone dependence, yet there are few studies on the association between breast disease and uterine fibroids (UFs). The purpose of this study was to investigate the incidence of benign breast disease (BBD), carcinoma in situ (CIS), and breast cancer (BC) in women treated for UFs compared to women who were not treated for UFs. This retrospective cohort study used national health insurance data from January 1st, 2011, to December 31st, 2020. We selected women between 20 and 50 years old who (1) were treated for UFs (UF group) or (2) visited medical institutions for personal health screening tests without UFs (control group). We analyzed independent variables such as age, socioeconomic status (SES), region, Charlson comorbidity index (CCI), delivery status, menopausal status, menopausal hormone therapy (MHT), endometriosis, hypertension (HTN), diabetes mellitus (DM), and dyslipidemia based on the first date of uterine myomectomy in the UF group and the first visiting date for health screening in the non-UF group. There were 190,583 and 439,940 participants in the UF and control groups, respectively. Compared with those of the control group, the RRs of BBD, CIS, and BC were increased in the UF group. The hazard ratios (HRs) of BBD, CIS, and BC in the UF group were 1.335 (95% confidence interval (CI) 1.299–1.372), 1.796 (95% CI 1.542–2.092), and 1.3 (95% CI 1.198–1.41), respectively. When we analyzed the risk of BC according to age at inclusion, UFs group had the increased risk of BCs in all age groups in comparison with control group. Women with low SES (HR 0.514, 95% CI 0.36–0.734) and living in rural areas (HR 0.889, 95% CI 0.822–0.962) had a lower risk of BC. Our study showed that women with UFs had a higher risk of BBD, CIS, and BC than those without UFs. This result suggests that women with UFs should be more conscious of BC than those without UFs. Therefore, doctors should consider recommending regular breast self-exams, mammography, or ultrasound for the early detection of BC in women with UFs.

## Introduction

Uterine fibroids (UFs) are noncancerous growths that develop in or around the uterus, sometimes called uterine leiomyoma or myoma^1^. UFs are the leading cause of hysterectomy in the United States^2^. The incidence of UFs increases with age from menarche to perimenopause and gradually decreases after menopause, signifying that estrogen and progesterone play prominent roles in promoting growth^3^. UFs may cause excessive uterine bleeding and subsequent anemia, pelvic discomfort, urinary incontinence, recurrent miscarriage, preterm labor, and infertility^4^. Marshall et al. reported that the incidence rates of UFs increased with age, and the age-standardized rates of ultrasound- or hysterectomy-confirmed diagnoses per 1000 woman-years were 8.9 among white women and 30.6 among black women^5^. By the time they reach 50 years of age, nearly 70% of white women and more than 80% of black women will have at least one UF^5^.

Although the etiology of UFs remains poorly understood, a large body of epidemiological, clinical, and experimental data indicates that reproductive factors, ovarian steroid hormones, and genetic and environmental factors play a role in the pathogenesis of UFs^6–9^. Numerous clinical, molecular, biological, and pharmacological studies have suggested that 17β-estradiol (E2) and progesterone (P4) play an important role in the development, growth, and maintenance of UFs^6^. However, the relative contributions of E2 and P4 to the pathogenesis of UFs are still controversial. Many researchers have consistently reported that estrogen and estrogen receptors are the main inducers of UF development^10–12^. Early menarche and obesity, which are risk factors for breast cancer, are believed to be associated with an increased incidence of UFs^13^. Similarly, estrogen and progesterone exposure are a significant risk factor for breast cancer^14^. The overexpression of estrogen receptors in normal breast epithelium may augment estrogen sensitivity and hence the risk of breast cancer^15^. Benign breast disease (BBD) and UFs are most commonly diagnosed in women with high circulating blood estrogen levels during the reproductive period^3, 16^. Peterson et al. reported that the human mammary gland containing ER-positive cells had a distribution of scattered single cells, with the highest frequency and intensity of measured staining in the lobules compared to the interlobular ducts, and an average of 87% of the ER-positive cells were luminal epithelial cells or occupied an intermediate position in the duct wall^17^.

Numerous clinical studies support the fact that BBD increases the risk of breast cancer (BC)^18, 19^. Hartman et al. reported that the relative risk of BC was 4.24 in women with atypical hyperplasia (AH), 1.88 in women with proliferative lesions (PLs), and 1.27 in women with non-proliferative lesions (NPLs). A family history of BC was a risk factor for BC independent of histological findings^18^. A population-based retrospective cohort study conducted in Spain showed that the risk of BC increased in women with proliferative or nonproliferative BBD regardless of their family history of BC^19^.

We designed a cohort study to investigate the incidence of BBD, CIS, and BC in women treated for UFs compared to women who had not UFs based on national cohort data in South Korea.

## Method

### Database

The retrospective cohort study was conducted using data from January 1st, 2011, to December 31st, 2020, provided by the Health Insurance Review and Assessment Service (HIRA). The National Health Insurance Service (NHIS) is a national insurance system run by the government of the Republic of Korea, and all Koreans (approximately 51 million) are obliged to join by law^20^. All healthcare use information is registered with the NHIS and HIRA. Thus, this database provides a vast amount of information, including demographic information, diagnostic codes, surgical codes, health insurance types, and prescription drugs, except in cases such as cosmetic surgery.

### Selection of participants

Diagnosis is classified according to the International Classification of Diseases, 10th revision (ICD-10) code. The surgical and examination codes are classified according to the Korea Health Insurance Medical Care Expenses (2016, 2019 version). The women with UFs between 20 and 50 years old who were treated with myomectomy (R4121, R4122, R4123, R4124, R4125, R4126, R4127, R4128, R4129) were extracted as the UF group. Then, we sorted outpatients with concurrent diagnostic codes of uterine fibroids D25.x as the primary or secondary diagnosis among myomectomy patients. The non-UF group was selected from women between 20 and 50 years old who visited medical institutions for personal health screening tests. The age group was divided into 20 s, 30 s, and 40 s. Among those selected for the non-UF group, women who had a UF diagnosis code in their chart were excluded. Women in either group with any cancer (C), any BBD (N60-63, D24), any CIS (D05), or any BC (C50) prior to inclusion were excluded from the study. Women excluded subjects who were diagnosed as any breast diseases between 2009 and 2010 from this study for washout period.

### Definition of outcome/event

We grouped all breast diseases into three subgroups; BBD (N60, N61, N62, N63, and D24), CIS (D05), and BC (C50) and analyzed incidence of breast diseases. The presence of any breast disease was defined as a case in which the patient visited a medical institution at least three times with the diagnostic codes BBD, CIS, or BC.

### Variables

We set independent variables such as age, socioeconomic status (SES), region, Charlson comorbidity index (CCI), delivery status (parity), menopausal status, menopausal hormone therapy (MHT), endometriosis, hypertension (HTN), diabetes mellitus (DM), and dyslipidemia based on the first date of uterine myomectomy in the UF group and the first visiting date for health screening in the control group. We categorized the age variable into five-year intervals between 20 and 50 years old. We categorized the type of medical insurance into low SES and mid/high SES according to whether women had been served with medical aid. We categorized the residence regions into urban and rural areas according to whether the women lived in metropolitan areas. The CCI was calculated using diagnostic codes from data during the year prior to study entry^21^.

We classified the parity according to whether women had a diagnosis code related to delivery (O80, O81, O82, O83, and O84). The patients in the study group were determined to be menopausal if the menopause diagnosis code (N95, M810, M800, and E238) was found more than twice in an individual.

### Statistics

All statistical analyses were performed using SAS Enterprise Guide 6.1 (SAS Institute Inc. Cary, North Carolina, USA) and R version 3.0.2 (The R Foundation for Statistical Computing, 2013, Vienna, Austria). The tests were two-sided, and a p value less than 0.05 indicated statistical significance. We used descriptive statistics with frequencies and percentages for categorical variables and median values (25th percentile, 75th percentile) for continuous variables. We used the *t* test and Mann‒Whitney *U* test for continuous parametric variables and the Pearson chi-square test and Fisher’s exact test for categorical variables.

To further understand the association between uterine fibroids and breast disease after adjustment for several different variables, we used the Cox proportional hazard model. The pairwise deletion method is performed if the missing value is less than 10%. The regression enhancement method is performed if the missing value is more than 10%.

### Ethics

This study was approved and waived informed consent by the IRB of Sanggye Paik Hospital (Approval number: SGPAIK-2021-02-005). This study have been performed in accordance with the Declaration of Helsinki. This study uses data provided by the HIRA, but the HIRA and the Ministry of Health and Welfare of Korea have no interest in this study. The HIRA has a data management policy in which all data resources should be provided to investigators as selective information that cannot identify individuals. Therefore, the researchers cannot identify individuals with the data used in the study. In addition, for personal information protection, the HIRA regulates raw data to be read only on HIRA’s server, and the result values (tables, pictures) can be taken out of the server. For this reason, this study does not need to provide informed content to participants in the study based on the Bioethics and Safety Act of South Korea.

## Results

**Figure 1:**
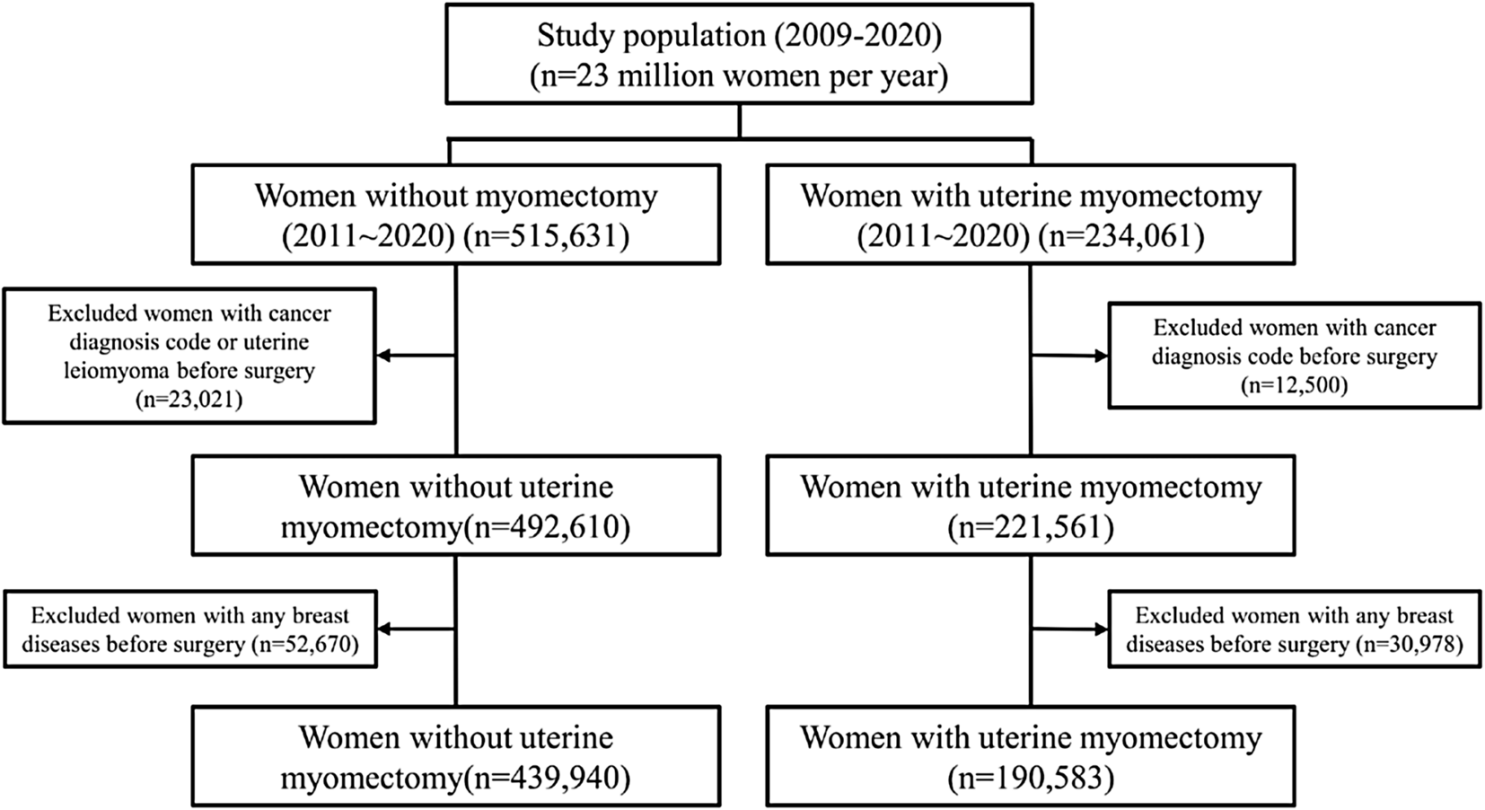
Flowchart for selecting case and control groups in this study using HIRA data. The UF group and the control group included 190,583 and 439,940 patients, respectively, from January 1st, 2011 to December 31st, 2020. *UF* uterine fibroid, *HIRA* The Health Insurance Review and Assessment Service.

The UF group and the control group included 190,583 and 439,940 patients, respectively, from January 1st, 2011 to December 31st, 2020 (Fig. 1). Detailed demographic characteristics of participants and the incidences of breast disease in participants with UFs group or control group and are shown in Table 1.

**Table 1:** Characteristics of participants and the cases of breast disease in participants with UFs group or control group in HIRA claim data.

	Control	UFs	Total	P-value*
Number of participants	439,940	190,583	630,523	
Median age (years)	34 [28–41]	40 [34–44]	36 [30–42]	< 0.001
Age at inclusion (years)				< 0.001
20 ~ 29	141,455 (32.2)	15,139 (7.9)	156,594 (24.8)	
30 ~ 39	167,901 (38.2)	77,792 (40.8)	245,693 (39)	
40 ~ 49	130,584 (29.7)	97,652 (51.2)	228,236 (36.2)	
SES low	9555 (2.2)	2116 (1.1)	11,671 (1.9)	< 0.001
Mid ~ high	430,385 (97.8)	188,467 (98.9)	618,852 (98.1)	
Region rural	209,799 (47.7)	71,990 (37.8)	281,789 (44.7)	< 0.001
Urban	230,141 (52.3)	118,593 (62.2)	348,734 (55.3)	
CCI 0	349,295 (79.4)	151,897 (79.7)	501,192 (79.5)	< 0.001
1	60,161 (13.7)	24,012 (12.6)	84,173 (13.3)	
≥ 2	30,484 (6.9)	14,674 (7.7)	45,158 (7.2)	
Parity 0	334,449 (76)	170,031 (89.2)	504,480 (80)	< 0.001
1	70,251 (16)	13,261 (7)	83,512 (13.2)	
≥ 2	35,240 (8)	7291 (3.8)	42,531 (6.7)	
Menopause	8799 (2)	3011 (1.6)	11,810 (1.9)	< 0.001
MHT	3106 (0.7)	1121 (0.6)	4,227 (0.7)	< 0.001
Endometriosis	9708 (2.2)	29,641 (15.6)	39,349 (6.2)	< 0.001
Hypertension	22,226 (5.1)	12,372 (6.5)	34,598 (5.5)	< 0.001
DM	19,759 (4.5)	8415 (4.4)	28,174 (4.5)	0.18
Dyslipidemia	57,198 (13)	27,497 (14.4)	84,695 (13.4)	< 0.001
BBD	13,923 (3.2)	10,865 (5.7)	24,788 (3.9)	< 0.001
CIS	332 (0.1)	439 (0.2)	771 (0.1)	< 0.001
BC	1360 (0.3)	1267 (0.7)	2,627 (0.4)	< 0.001

*BBD* benign breast disease, *BC*, breast cancer; *CCI* Charlson comorbidity index, *CIS* carcinoma in situ, *DM*, diabetes mellitus, *MHT* menopausal hormone therapy, *SES* socioeconomic status, *UFs* uterine fibroids, *HIRA* health insurance review & assessment Service, *UFs* uterine fibroids.

Data are expressed as the number (%) or median [25 percentile–75 percentile].

^*^p-value less than 0.05 is statistically significant.

**Figure 2:**
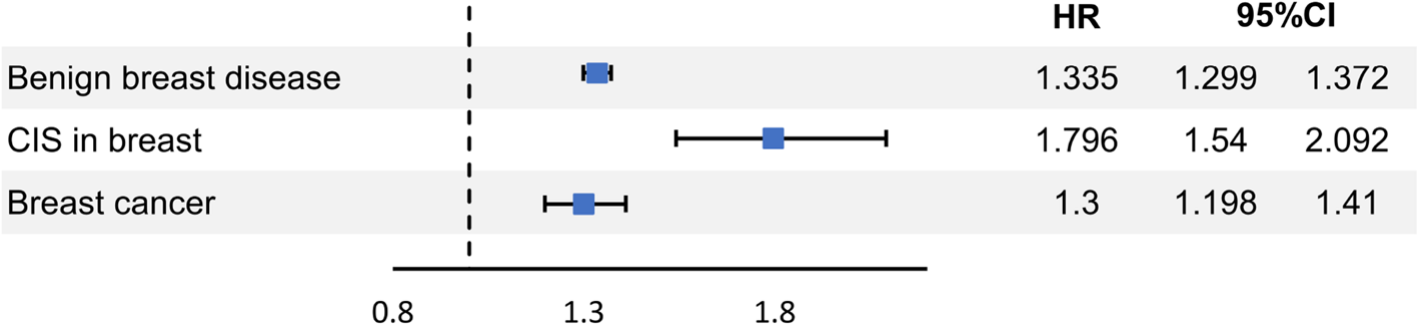
Hazard ratios of breast diseases in the uterine fibroid group. The hazard ratios (HRs) of BBD, CIS, and BC in the UF group were 1.335 (95% confidence interval CI 1.299–1.372), 1.796 (95% CI 1.542–2.092), and 1.3 (95% CI 1.198–1.41), respectively. *BBD* benign breast disease, *CIS*, carcinoma in situ, *BC* breast cancer.

The incidences of BBD were 10,865 (5.7%) in the UFs group and 13,923 (3.2%) in the control group (*p*-value < 0.001). The incidences of CIS were 439 (0.2%) in the UFs group and 332 (0.1%) in the control group (*p*-value < 0.001). The incidences of BC were 1267 (0.7%) in the UFs group and 1360 (0.3%) in the control group (*p*-value < 0.001). (Table 1). The hazard ratios (HRs) of breast diseases in participants with/without UFs according to independent variables such as SES, living area, CCI, parity, menopausal status, MHT, endometriosis, HTN, DM, and dyslipidemia were presented in the Table 2. The HRs of BBD, CIS, and BC in the UF group were 1.335 (95% confidence interval CI 1.299–1.372), 1.796 (95% CI 1.542–2.092), and 1.3 (95% CI 1.198–1.41), respectively (Fig. 2). The HRs of breast diseases in participants with UFs in comparison with non-UFs group statistically significantly increased in all age groups except the HR of BBD in the 30 s (Table 2).

**Table 2:** Hazard ratios of breast diseases in participants with/without UFs.

	BBD		CIS in breast		BC	
	HR (95% CI)^a^	*p*-value	HR (95% CI)^a^	*p*-value	HR (95% CI)^a^	*p*-value^†^
UFs (reference = non-UFs)	1.335 (1.299–1.372)	< 0.001	1.796 (1.542–2.092)	< 0.001	1.3 (1.198–1.41)	< 0.001
20 ~ 29 years	1.365 (1.244–1.497)	< 0.001	1.287 (1.234–1.341)	< 0.001	1.363 (1.31–1.419)	< 0.001
30 ~ 39 years	2.077 (0.884–4.879)	0.094	1.831 (1.431–2.341)	< 0.001	1.751 (1.436–2.136)	< 0.001
40 ~ 49 years	2.767 (1.754–4.366)	< 0.001	1.36 (1.181–1.565)	< 0.001	1.219 (1.101–1.35)	< 0.001
Age (years) (reference = 20 ~ 29)						
30 ~ 39	1.532 (1.474–1.593)	< 0.001	4.342 (3–6.284)	< 0.001	4.71 (3.823–5.803)	< 0.001
40 ~ 49	1.568 (1.507–1.632)	< 0.001	6.736 (4.673–9.711)	< 0.001	8.835 (7.195–10.848)	< 0.001
Low SES	0.647 (0.579–0.723)	< 0.001	0.501 (0.249–1.008)	0.053	0.514 (0.36–0.734)	< 0.001
Region (rural area)	0.836 (0.815–0.858)	< 0.001	0.743 (0.639–0.862)	< 0.001	0.889 (0.822–0.962)	0.004
CCI						
1	1.089 (1.05–1.129)	< 0.001	1.055 (0.858–1.298)	0.611	1.013 (0.905–1.135)	0.821
≥ 2	1.128 (1.078–1.181)	< 0.001	1.119 (0.869–1.441)	0.383	1.17 (1.023–1.339)	0.022
Parity in cohort (reference = 0)						
1	0.884 (0.846–0.924)	< 0.001	0.912 (0.688–1.209)	0.522	0.993 (0.858–1.15)	0.928
≥ 2	0.868 (0.816–0.924)	< 0.001	0.813 (0.541–1.223)	0.321	1.108 (0.918–1.338)	0.286
Menopause	1.145 (1.054–1.244)	0.001	0.956 (0.603–1.516)	0.849	0.877 (0.688–1.118)	0.291
MHT	0.986 (0.836–1.163)	0.865	0 (0-infinite)	0.93	0.515 (0.254–1.044)	0.066
Endometriosis	1.157 (1.105–1.212)	< 0.001	1.131 (0.887–1.442)	0.322	1.024 (0.885–1.186)	0.746
Hypertension	0.905 (0.854–0.959)	< 0.001	0.989 (0.735–1.331)	0.942	0.932 (0.793–1.094)	0.388
DM	0.96 (0.899–1.025)	0.224	0.884 (0.607–1.287)	0.52	0.994 (0.822–1.203)	0.954
Dyslipidemia	1.055 (1.013–1.098)	0.009	1.014 (0.809–1.271)	0.906	1.017 (0.901–1.147)	0.789

*BBD* benign breast disease, *BC* breast cancer, *CCI* Charlson comorbidity index, *CI* confidence interval, *CIS* carcinoma in situ, *DM* diabetes mellitus, *HR* hazard ratio, *MHT* menopausal hormone therapy, *SES* socioeconomic status, *UFs* uterine fibroids.

^a^HRs were adjusted for uterine fibroid, age, SES, regrion, CCI, parity, menopause, MHT, endometriosis, hypertension, DM, dyslipidemia.

^†^*p*-value less than 0.05 is statistically significant.

**Figure 3:**
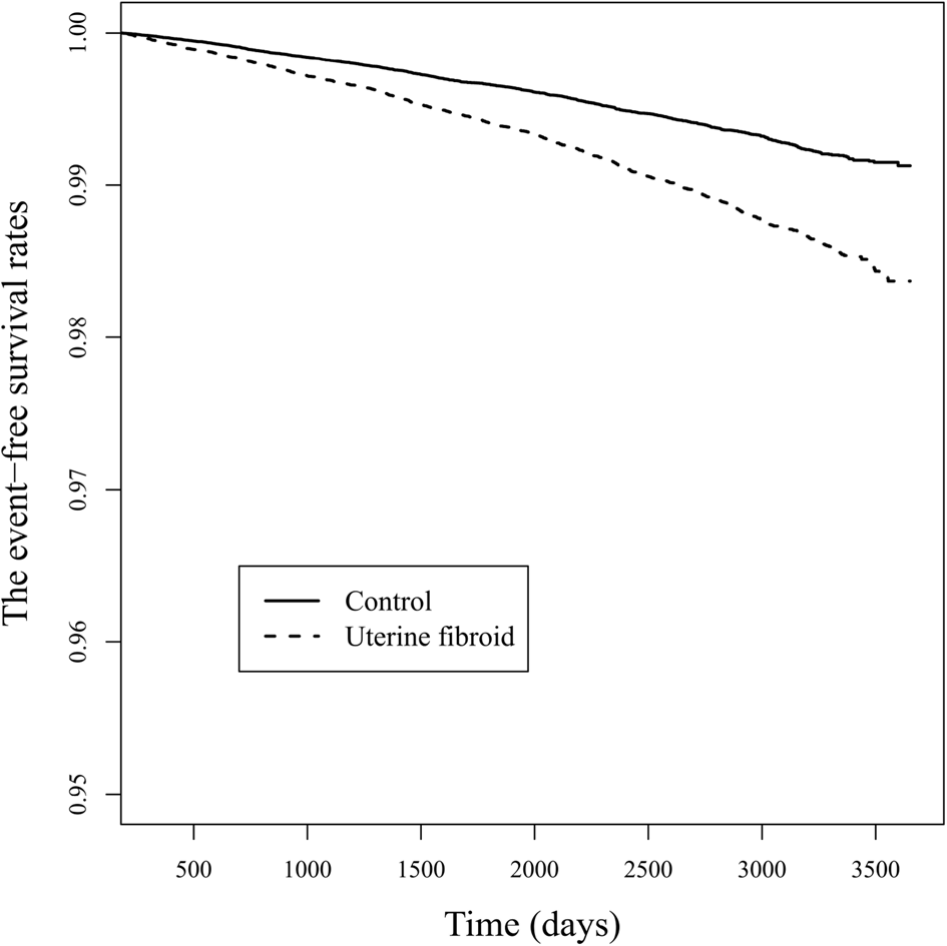
The event -free survivals of breast cancer in uterine fibroid group and control group.

The HRs of all-cause mortality in participants with BC was 1.001 (95% CI 0.534–1.877) in the UFs group (Fig. 3). The only significant variable related with increased mortality of breast cancer was parity 2 or more (HR 3.545, 95% CI 1.171–10.731) (*p*-value = 0.025) (Table 3). The incidence of BC per 100,000 person-years was 1360 in participants with UFs and 1267 in participants without UFs; the details are presented in Supplementary Table 1. In the sensitivity test, the HR of breast cancer was statistically significantly higher in the UFs group than in the control group (HR 1.295, 95% CI 1.189–1.410, *p*-value < 0.001) (Supplementary Table 2).

**Table 3:** Hazard ratios of all-cause mortality in participants with breast cancer.

	BC	
	HR (95% CI)^a^	*p*-value^†^
UFs	1.001 (0.534–1.877)	0.998
Age (years) (reference = 20 ~ 29)		
30 ~ 39	0.518 (0.171–1.566)	0.244
40 ~ 49	0.598 (0.2–1.791)	0.359
Low SES	0 (0-infinite)	0.993
Region (rural area)	1.338 (0.737–2.429)	0.339
CCI		
1	0.268 (0.064–1.117)	0.071
≥ 2	0.601 (0.18–2.007)	0.408
Parity in cohort (reference = 0)		
1	1.94 (0.704–5.347)	0.2
≥ 2	3.545 (1.171–10.731)	0.025
Menopause	0 (0-infinite)	0.989
MHT	0 (0-infinite)	0.987
Endometriosis	1.074 (0.319–3.614)	0.909
Hypertension	0.429 (0.058–3.175)	0.408
DM	0.683 (0.089–5.257)	0.714
Dyslipidemia	0.726 (0.215–2.445)	0.605

*BC* breast cancer, *CCI* Charlson comorbidity index, *CI*, confidence interval, DM diabetes mellitus, *HR* hazard ratio, *MHT* menopausal hormone therapy, *SES* socioeconomic status, *UFs* uterine fibroids.

^a^HRs were adjusted for uterine fibroid, age, SES, regrion, CCI, parity, menopause, MHT, endometriosis, hypertension, DM, dyslipidemia.

^†^*p*-value less than 0.05 is statistically significant.

## Discussion

This study presented that the risks of BBD, CIS, and BC were higher in the women who had undergone surgery for symptomatic UFs than in the control. Several previous reports showed an increased BC risk in women with UFs^22–24^. A previous study reported that women with a history of UFs were at increased risk of BC^22^. However, their study has several limitations. First, it is a population-based case‒control study that the prevalence of UFs was higher in women with BC than in women without BC. We should be careful to discuss the causal relationship between BC and the prevalence of UFs because their study excluded the temporal concept of each event. Second, their study included both asymptomatic and symptomatic cases of UFs regardless of hysterectomy. This wide inclusion criteria could decrease the accuracy of the exact relations of pathologic UFs with BC. Third, their study did not analyze any other outcomes like BBD and carcinoma in situ (CIS) except BC and also did not present the data of the follow-up period.

Conversely, the results of the Black Women’s Health Study (BWHS) of the US suggest that a history of UF diagnosis is unrelated to the risk of BC overall. However, positive associations were observed for early diagnosed UFs with young age BC before age 40^25^.

Among the many causes of the strong association between UFs and BC, the first thing researchers noticed was female sex hormones. UFs depend on ovarian estrogen and progesterone, which are essential for UF growth, and most UFs shrink after menopause^12^. Gonadotropin-releasing hormone (GnRH), which suppresses ovarian function and reduces circulating levels of estrogen and progesterone, is sometimes used to treat UFs^26^. Sharp elevations and declines in the production of estrogen and progesterone are observed during very early pregnancy and the postpartum period, which have a dramatic effect on UF growth^27^. On the other hand, the WHI study presented strong evidence for the carcinogenic role of progesterone and its agonists, which indicated that treatment with the estrogen plus progestin combination increased postmenopausal BC risk, whereas estrogen alone did not^14^. Additionally, a new genome-wide association study (GWAS) meta-analysis reported a significant genetic correlation of UFs with BC, especially a possible causal link with estrogen receptor (ER)-positive BC. They suggested that those findings indicate an intrinsic link underlying UFs and BC^28^.

The other explanation for the association between UFs and BC could be that they share many nonmodifiable and modifiable risk factors. The risk factors for UFs have been identified as race (black), age (over 40 years), family history, menstrual history, endogenous hormonal levels, time since last birth, premenopausal status, physical activity and body mass index (BMI), lifestyle and diet, stress, endocrine-disrupting chemicals (EDCs), oral contraceptives, etc.^5, 29–33^. Similar to UFs, the risk of BC is also determined by complex mechanisms involving an individual’s genetic, physiological, reproductive, lifestyle, and environmental factors^34^, which include age, race, obesity, MHT, environmental pollutants, and EDCs^35–37^.

Furthermore, certain estrogen-mimicking EDC (EED) exposures could potentially elevate the risk of UFs and BC at the same time^38^. EEDs such as polychlorinated biphenyls (PCBs), bisphenol A (BPA), and phthalates are ubiquitous substances that are found in products that are used in our everyday lives, including pesticides, plasticizers, pharmaceutical agents, personal care products, fungicides, herbicides, cosmetics, food products and food packaging. Exposure to EDCs is potentially carcinogenic as it can cause epigenetic modifications, thus increasing the risk for UFs and BC^38, 39^.

One of the essential results of our study is that the risk of BBD was higher in women treated with symptomatic UFs than in the control group. Although BBD and UFs are most commonly diagnosed in women in the reproductive period, there have been no previous studies about the association between UFs and BBD, such as ours. BBD has been directly or indirectly associated with lifetime sex steroid hormone exposure in various epidemiological studies^40^. As mentioned in the introduction, a population-based retrospective case‒control study showed that the risk of BC in women with proliferative or nonproliferative BBD increased regardless of family history^19^. A meta-analysis showed that the risk of BC gradually increased with various probabilities according to tissue diagnoses, such as NPL, PL, and AH^41^. The higher incidence of BBD in women with UFs leads to higher BC risk factors, so it is a predictable result that both BBD and BC were higher in women treated for symptomatic UF group than in the control group.

Looking more closely at our results, the women with low SES and living in the rural area had a lower incidence of BC than those with high SES and living in the metropolitan area. A case‒control study reported that high SES and hormone therapies significantly affected BC risk, and that white collar workers had a significantly higher risk of BC compared to manual workers regardless of menopausal status^42^. A systematic literature review mainly found consistent evidence that the risk of BC continues to be higher in higher SES group than in lower SES group. However, despite their conclusion, those results were confined to only 39 out of 55 papers^43^. Many studies have been conducted to analyze the risk of BC according to SES levels, but there is still much controversy. It is impossible to evaluate SES levels as a unified criterion because socioeconomic structures and systems, cultural characteristics, and women’s social and traditional roles vary among populations in different research studies. Unlike other studies, the SES groups in our study were divided according to whether women had been served with medical aid, yet the results were still consistent with the majority of previous studies.

Our study presented that the women with high score of CCI and no parity were associated with the risk of BBD than their counterpart, but were not associated with the risk of CIS and BC. Our study also presented that the risk of BBD was higher in women with endometriosis than in women without endometriosis, but the risk of CIS or BC was not. Endometriosis has been known to be associated with a modestly increased risk of both proliferative and nonproliferative BBD^44^. This finding is inconsistent with the majority of previous studies reporting an increased risk of BC in women with endometriosis. A preliminary study suggests that the upregulation of inflammatory and hormonal mediators is common between endometriosis and BC^45^. Although the majority of the studies supported an association between the two, issues regarding the association between endometriosis and BC risk are still inconclusive^46, 47^. A study reported that the overall risk of BC in women with surgically verified endometriosis was similar to that in the general population^48^. We do not know the exact mechanisms involved in an inverse association between endometriosis and BC, but Matta et al. suggested that higher DNA repair capacity (DRC) in women with endometriosis and/or hormonal treatments for endometriosis may provide specific protective effects for BC^49^. Additionally, progesterone’s pleiotropic and complex actions are evident in the breast and uterus. Even within the same uterus, progesterone stimulates the growth of leiomyomas but inhibits the growth of the endometrium. The paracrine interactions of PR-expressing stroma represent a critical difference between the endometrium and myometrium. In contrast, the primary target of progesterone is mammary epithelial cells in the breast and leiomyoma cells in fibroids, which lack specifically organized stromal components with significant PR expression^50^.

The strength of our study is that it is the first study on the association between BBD and UFs and is consistent with previous studies showing that women with BBD had a higher risk of BC. In addition, we found that the risk of BBD and BC in women with UFs increased simultaneously. However, this study has some limitations. First, although adjustments were made for the numerous factors related to the occurrence of BC, we must be careful in the interpretation of our results because this study has the limitation of retrospective cohort studies. Second, our study did not include women with asymptomatic UFs or women with mild symptoms who did not have surgical indications from the beginning. Therefore, we have to be concerned about the possibility that the exact incidence of symptomatic UFs has been underestimated, which suggests the need for further research to analyze the incidence of breast disease, including patients with asymptomatic UFs.

## Conclusion

Our study showed that women who had surgery for symptomatic UFs had a higher risk of BBD, CIS, and BC than those who did not have UFs. This result indicates that women with symptomatic UFs should be more conscious of breast cancer than women without symptomatic UFs. Therefore, doctors should consider recommending regular breast self-exams, mammography, or ultrasound for the early detection of breast cancer in women with symptomatic UFs.

### Supplementary Information

Supplementary Tables.

## Data Availability

All data generated or analysed during this study are included in this published article and its supplementary information files. The datasets generated and/or analysed during the current study are not publicly available. This is because the dataset for this study is only available on the NHIS servers for one year after the dataset was generated. Therefore, the data of the series will not be available for sharing by bona fide researchers or for further statistical analysis in the future. However, upon reasonable request, the corresponding author will consider a response to explain the details of the data.

## Abbreviations

AH: Atypical hyperplasia
BBD: Benign breast disease
BC: Breast cancer
BMI: Body mass index
BPA: Bisphenol A
BWHS: Black women’s health study
CCI: Charlson comorbidity index
CI: Confidence interval
CIS: Carcinoma in situ
DM: Diabetes mellitus
DRC: DNA repair capacity
EDCs: Endocrine-disrupting chemicals
EED: Estrogen-mimicking EDC
E2: 17β-Estradiol
P4: Progesterone
GWAS: Genome-wide association study
HIRA: Health insurance review and assessment Service
HR: Hazard ratio
HTN: Hypertension
ICD-10: International classification of diseases, 10th revision
IRB: Institutional review board
MHT: Menopausal hormone therapy
NHIS: National health insurance service
NPLs: Non-proliferative lesions
PCBs: Polychlorinated biphenyls
PLs: Proliferative lesions
RCT: Randomized clinical trial
SES: Socioeconomic status
WHI: Women’s health initiative
UFs: Uterine fibroids
