# Supplementary material for: Association between breast diseases and symptomatic uterine fibroids by using South Korean National Health Insurance database
Source: Sci Rep. 2023 Oct 5;13:16772. doi: 10.1038/s41598-023-43443-w (PMC10555995; doi:10.1038/s41598-023-43443-w)
Supplement: Supplementary file 1 — Supplementary Tables. [file 41598_2023_43443_MOESM1_ESM.docx]

Supplementary Table 1. Case/person-years of breast cancer in participants with UFs and control group.

|  |  |  | |  | |
| --- | --- | --- | --- | --- | --- |
|  | Control | UFs | | |  |
| Total | 1,360/1,839,131 (74) | 1,267/942,247 (134) |  | |  |
| Age at inclusion (years) |  |  |  | |  |
| 20~29 | 73/547,392 (13) | 27/76,380 (35) |  | |  |
| 30~39 | 517/733,762 (70) | 412/390,744 (105) |  | |  |
| 40~49 | 770/557,978 (138) | 828/475,123 (174) |  | |  |
| SES |  |  |  | |  |
| Mid~high SES | 1,333/1,794,699 (74) | 1,263/931,099 (136) |  | |  |
| Low SES | 27/44,432 (61) | 4/11,148 (36) |  | |  |
| Region |  |  |  | |  |
| Urban area | 731/986,918 (74) | 830/596,805 (139) |  | |  |
| Rural area | 629/852,213 (74) | 437/345,442 (127) |  | |  |
| CCI |  |  |  | |  |
| 0 | 1,042/1,451,521 (72) | 986/746,513 (132) |  | |  |
| 1 | 189/251,431 (75) | 164/120,388 (136) |  | |  |
| ≥2 | 129/136,180 (95) | 117/75,346 (155) |  | |  |
| Parity in cohort |  |  |  | |  |
| 0 | 1,092/1,445,389 (76) | 1,196/871,640 (137) |  | |  |
| 1 | 171/271,082 (63) | 47/47,775 (98) |  | |  |
| ≥2 | 97/122,661 (79) | 24/22,831 (105) |  | |  |
| Menopause |  |  |  | |  |
| No | 1,309/1,795,274 (73) | 1,247/926,771 (135) |  | |  |
| Yes | 51/43,858 (116) | 20/15,476 (129) |  | |  |
| MHT |  |  |  | |  |
| No | 1,353/1,827,875 (74) | 1,266/938,148 (135) |  | |  |
| Yes | 7/11,256 (62) | 1/40,099 (2) |  | |  |
| Endometriosis |  |  |  | |  |
| No | 1,334/1,803,914 (74) | 1,087/804,592 (135) |  | |  |
| Yes | 26/35,217 (74) | 180/137,655 (131) |  | |  |
| Hypertension |  |  |  | |  |
| No | 1,271/1,751,338 (73) | 1,185/885,338 (134) |  | |  |
| Yes | 89/87,793 (101) | 82/56,909 (144) |  | |  |
| DM |  |  |  | |  |
| No | 1,296/1,766,686 (73) | 1,212/905,299 (134) |  | |  |
| Yes | 64/72,446 (88) | 55/36,948 (149) |  | |  |
| Dyslipidemia |  |  |  | |  |
| No | 1,189/1,644,870 (72) | 1,109/829,880 (134) |  | |  |
| Yes | 171/194,262 (88) | 158/112,367 (141) |  | |  |
| CCI, Charlson comorbidity index; DM, diabetes mellitus; MHT, menopausal hormone therapy; SES, socioeconomic status; UFs, uterine fibroids | | |  | |  |
| Data are expressed as the case/person-years (case/ 100,000 person-years). | | |  | |  |

Supplementary table 2. Sensitivity test. Hazard ratios of breast diseases in participants with UFs and control group.

|  |  |  |  |  |  | |  |
| --- | --- | --- | --- | --- | --- | --- | --- |
|  | BBD | | CIS | | | BC | |
|  | HR (95% CI) ^a^ | P-value | HR (95% CI) ^a^ | P-value | HR (95% CI) ^a^ | | P-value |
| UFs | 1.3 (1.263-1.338) | <0.001 | 1.779 (1.518-2.085) | <0.001 | 1.295 (1.189-1.41) | | <0.001 |
|  |  |  |  |  |  | |  |
| BBD, benign breast disease; BC, breast cancer; CIS, carcinoma in situ**;** CCI, Charlson comorbidity index; CI, confidence interval; DM, diabetes mellitus; HR, hazard ratio; MHT, menopausal hormone therapy; SES, socioeconomic status; UFs, uterine fibroids | | | | | | | |
| ^a^ HRs were adjusted for uterine fibroid, age, SES, regrion, CCI, parity, menopause, MHT, endometriosis, hypertension, DM, dyslipidemia. | | | | | | | |
